# Information theory-based algorithm for *in silico *prediction of PCR products with whole genomic sequences as templates

**DOI:** 10.1186/1471-2105-6-190

**Published:** 2005-07-26

**Authors:** Youfang Cao, Lianjie Wang, Kexue Xu, Chunhai Kou, Yulei Zhang, Guifang Wei, Junjian He, Yunfang Wang, Liping Zhao

**Affiliations:** 1 School of Life Science and Biotechnology, Shanghai Jiao Tong University, Shanghai 200240, China; 2 Department of Food Science and Engineering, Northwest Institute of Light Industry, Xianyang 712081, Shaanxi, China; 3 Department of Computer Science, Shanghai Jiao Tong University, Shanghai 200240, China; 4 Institute of Botany, Chinese Academy of Sciences, Beijing 100093, China

## Abstract

**Background:**

A new algorithm for assessing similarity between primer and template has been developed based on the hypothesis that annealing of primer to template is an information transfer process.

**Results:**

Primer sequence is converted to a vector of the full potential hydrogen numbers (3 for G or C, 2 for A or T), while template sequence is converted to a vector of the actual hydrogen bond numbers formed after primer annealing. The former is considered as source information and the latter destination information. An information coefficient is calculated as a measure for fidelity of this information transfer process and thus a measure of similarity between primer and potential annealing site on template.

**Conclusion:**

Successful prediction of PCR products from whole genomic sequences with a computer program based on the algorithm demonstrated the potential of this new algorithm in areas like *in silico *PCR and gene finding.

## Background

PCR technique is widely used in the molecular biology laboratory. The key step to successful PCR is primer design. Usually, software for PCR primer design only gives a subset of candidates, and we must choose the workable primers empirically. With more organisms' genomes sequenced and data freely available, we may predict PCR products with computer programs and evaluate primer candidates against the target organisms' genomic sequence prior to performing the PCR in the laboratory.

The most important thing for PCR product prediction is finding appropriate primer annealing sites on the template. Many attempts have been made to develop computer programs based on different algorithms for this purpose. Such programs include Amplify [1], simPCR [2], PCRAna [3], PUNS, and Virtual PCR [4]. PUNS [5] is a web-based program for PCR prediction, however, it does not deal with degenerate PCR primer. Some algorithms for the selection of probes or DNA oligos are also related to this field, such as longest common factor approach proposed by Rahmann [6], hybridization free energy based method used by Li [7], Kaderali's work [8] based on an extended nearest neighbor model, and Lexa's PRIMEX [9].

In our research, we designed a new algorithm based on information theory. One information source was obtained by converting the primer sequences to numeric vectors of the potential full hydrogen bond numbers, and the second was created as a vector of the actual hydrogen bond numbers formed between the primer and its potential binding site on the template. An information coefficient was computed for determining the similarity between the two information sources as a criterion to locate primer-annealing sites, and predict products. A computer program, SPCR (Simulated PCR), based on this algorithm was developed to predict PCR products, and its performance was evaluated by replicating 4 cases of laboratory PCR experiments *in silico*, and performing comparisons between the predicting results of our program and VPCR.

## Implementation

The algorithm is based on base digitalization followed by calculation of information coefficient. Information coefficient we used in this research is a formula based on Shannon's information theory. Shannon's information theory was ever used in other research concerned on primer design, e.g. Purohit et al. [10] used entropy measure to identify conserved regions in aligned sequences for primer design.

The first step of this algorithm is to find out appropriate annealing sites from template sequences. Firstly, let the upstream and downstream primer sequences slide along template sequences respectively, one base per step. At each position where 3'-end base of primers match with the base of templates, a DNA fragment can be acquired from template sequences which length is the same as primer length, which we name as Candidates of Annealing Sites (CAS). The primer sequences and CAS fragments are all converted into numeric vectors. We name the numeric vectors of two primer sequences as *PU *and *PD*, and fragments of template as *TU *and *TD*. They are defined as below.

*PU *= [*p*_1_, *p*_2_,..., *p*_*m*_], *PD *= [*p*_1_, *p*_2_,..., *p*_*n*_]     (1)

*TU *= [*t*_1_, *t*_2_,..., *t*_*m*_], *TD *= [*t*_1_, *t*_2_,..., *t*_*n*_]     (2)

Variables *m *and *n *are lengths of upstream and downstream primers. The *p*_*i *_and *t*_*i *_are defined as below.





According to equation (1), (2), (3) and (4), we can transform the DNA sequences of primers and CASs into numeric vectors. Then we can perform the next step, computing the information coefficient (*I*) for each primer-CAS pair. The formula for the calculation of information coefficient (*I*) is as equation (5).

Only those sites where the similarity is higher than a preset threshold were selected as annealing sites if the last digit in the vector of T was not 0 (a requirement for perfect match at the 3'-end).

The formula for information coefficient (*I*) calculation is as follows:



Here 

The value field of information coefficient (*I*) is (0,1], when primer sequence match with template completely, i.e. *P *= *T*, *I *= 1, and the higher the affinity between primer and template, the greater the value of information coefficient. Information coefficients formed by upstream primer and CAS of template are represented as a set *I*_*up*_; and accordingly, information coefficients of downstream primer and CAS are *I*_*dn*_.

For each probable product, a successful amplification was determined by 5 parameters: upstream information coefficient *I*_*up*_, downstream information coefficient *I*_*dn*_, estimated limits for product maximum and minimum length, and product amplification coefficient (*P*_*a*_) which equals to an average of *I*_*up *_and *I*_*dn*_. There are two kinds of average methods provided in SPCR program, the arithmetic average, i.e. (*I*_*up*_+*I*_*dn*_)/2, and the geometric average, (*I*_up_*I_*dn*_). We discuss only the geometric average here. Although the two method of average are different in computation, the values are close, and will not change the result of prediction significantly. If, and only if, the values of *I*_*up*_, *I*_*dn*_, and *P*_*a *_are all greater than the preset thresholds, and the length of predicted product lies in the preset length limit, did SPCR generate a product between upstream and downstream primer annealing sites within the product length range.

SPCR was implemented as a Win32 application and written in C++ language. It comprises an executable program that can be run directly without the need for installation. The user inputs the primer sequences, sets the thresholds, and provides locally one or more template sequence files, and push the button "Start PCR" to begin the prediction. SPCR can recognize degenerate primers encoded with the IUPAC nucleotide codes. Degenerated base are allowed in primer sequences. Template sequences can be available genomic sequence of the target organism, which must be Single- or Multi-FASTA format. The output of SPCR is a text file of a list of all predicted PCR products.

SPCR saves all produced data, including predicted products and all parameters, into a user-specified result file in pure text format. The output file consists of four parts. The first part is a table in which all of predicted products are listed, including their *P*_*a *_value, product length, template it comes from, direction of amplification, position of beginning and end, *I*_*up *_and *I*_*dn *_value, and the upstream and downstream primer sequences. The second part is a digit indicating the number of predicted products. The third part is the detailed nucleotide sequence data in FASTA format of all predicted products in the same order as in the table. The last part includes all parameters set before SPCR running. The time complexity of this algorithm is *O(n)*, where *n *is the aggregate length of all the template sequences. In addition, SPCR provides a function to simulate agarose gel patterns of output data.

## Results

To test the performance of the SPCR program, we first used SPCR to simulate the PCR experiments presented in the Virtual PCR (VPCR) paper [4].

**Figure 1:**
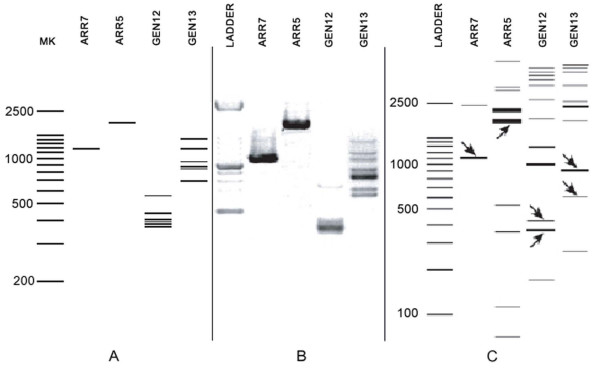
**The comparison of SPCR prediction results with real experimental results and Virtual PCR results**. Use of specific primers ARR5 (ARR5a, ARR5b), ARR7 (ARR7a, ARR7b), and degenerate primers (GEN12 and GEN13) to predict PCR products from the *Arabidopsis *genome. (A) Prediction results of SPCR, lanes ARR7 and ARR5 are ARR7 and ARR5 gene, respectively, amplified from the genome with the specific primers, lanes GEN12 and GEN13 are predicted results with the degenerate primers; (B) the laboratory PCR results; (C) prediction results of VPCR. Arrows show bands present on gels of actual PCR products. (B and C are adopted from Lexa et al. (2001) with permission.)

The SPCR prediction results for ARR5 and ARR7 genes were identical to the laboratory PCR results, and no unspecific products were produced (Fig. 1). VPCR gave one unspecific product for the ARR7 gene and 8 unspecific products for ARR5. The SPCR prediction for GEN12 and GEN13 families with degenerate primers gave a predicted agarose profile similar to the laboratory results. More than 80% of the predicted products had counterparts in the laboratory PCR results. VPCR predicted only 2 products for each of the gene families and gave more than 8 unspecific products in each case. Although Lexa et al. has released the 2.0 version of VPCR , the predictions of GEN12 and GEN13 are still not as accurate as SPCR.

We used the universal primer pair P0 and P6 [11] to predict 16S rRNA genes from 59 completed bacterial genomes. We correctly located all copies of 16S rRNA genes from 52 of the tested genomes with all threshold parameters at 0.85. Predictions for three strains yielded 1 or 2 unspecific products, which were eliminated when the threshold parameters were increased to 0.86 or 0.9. Discrepancies between SPCR prediction and GenBank data for three strains were found to be a result of incorrect annotation of the data. In only one strain was an unspecific product found that was longer than the expected gene copy.

SOX proteins contain a conserved HMG-related DNA binding domain, which shares at least 50% identity with that of SRY. Cremazy [12] designed a pair of highly degenerate primers, which were capable of amplifying a broad spectrum of SOX HMG sequences. We predicted SOX HMG sequences from *Homo sapiens *genome with the primer pair P5-2 and P3-1 and verified with the BLAST (blastn) program [13] that the top 10 products were all known SOX genes.

Two primer pairs for amplification of conserved regions in polymerase coronavirus genes were used to predict PCR products from 7 complete coronavirus genomes, including SARS-CoV and 14 other coronavirus species. All predictions yielded the expected products from all templates, 453 bp for primer pair IN-2(+)/IN-4(-) [14] and 251 bp for 2 Bp/4Bm [15]. Phylogenetic trees based on the predicted products were in agreement with the current taxonomy of coronaviruses.

Besides above cases, we also tested SPCR with successful prediction of primer pairs for genome-specific amplification of environmental bacteria. Unspecific PCR primers against bacterial genomes for random amplification of bacterial community samples also yielded satisfactory results.

The threshold value of 3 parameters *I*_*up*_, *I*_*dn *_and *P*_*a *_are selected empirically. Increase of the parameter values can lead to reduction of unspecific products. This corresponds to increase of annealing temperature for reducing unspecific products in actual PCR. Usually, the 3 parameters should be greater than 0.8, if a specific PCR is predicted. If parameters higher than 9.0 still give unspecific products we suggest change of primer pairs if specificity is a major concern.

## Discussion

Amplification of non-targeted products is a common problem in PCR experiments, especially when using complex templates, such as whole genomic DNA or mixtures of genomes. It has been suggested that mismatch tolerance during primer annealing to template is the most important reason for unspecific PCR products, followed by primer length, template size, and product size limits [2]. The current PCR prediction methods are mainly based on probabilistic theories for similarity analysis between primer and template, while some other methods are also used in this field, such as statistical thermodynamics [16] and string comparisons.

A successful computer program for PCR product prediction should be able to identify all potential annealing sites. Sequence similarity between primer and template is the primary factor for selecting annealing sites. In this work we developed a new algorithm to assess the similarity between primer and template after the base sequences were converted into vectors of hydrogen bond numbers. We consider annealing of primer to template a means of information transfer. The hydrogen bond number vector for the primer is the source information, while the vector for template is the target information. The difference between the two information sources is a reflection of the fidelity of this information transfer process. Since hydrogen bonds are formed as a result of specific base recognition between the two DNA strands and the total number of hydrogen bonds is also a major force holding the two strands together, the similarity between these two information sources may be a good estimate of the probability of both annealing site selection and annealed structure stability. The information coefficient calculated in this work is a measure of similarity between two information sources. The value varies between 0–1, reflecting complete difference to 100% identity. In the SPCR program, the threshold can be increased to reduce expected products, which is comparable to increasing annealing temperature to reduce unspecific products in laboratory PCR. In contrast to BLAST, this algorithm tolerates any type of mismatch between primer and template. The successful prediction of all copies of 16S rRNA genes in complete genomes demonstrates the potential of using this algorithm for gene prediction of newly sequenced genomes.

## Conclusion

In our evaluating cases, the SPCR program is reasonably good for predicting all potential PCR products with complex templates. This can help the user choose the primer pair that gives the least possible non-targeted products. However, the prediction for random PCR products with SPCR is not satisfactory. When the template is too big, as the case with the human genome, the running time can reach 48 hours.

In current version, SPCR do not consider the situation of insertions and deletions of template sequences, these situations should be considered in future versions. Some refinements for the algorithm can also be done in future, for example, considering the effect of base stacking and alternative penalty for mispairs may improve the accuracy of prediction.

## Availability and requirements

The SPCR program and supplemental materials, including details of all the prediction experiments in this paper are freely available at our website: , also see [Additional file 1]. SPCR program was developed with C++ under Win32 and Linux, so it can be run under both platforms. There is no restriction for using the SPCR program.

## Authors' contributions

YC developed the original SPCR program, tested it and analyzed the results of SPCR predictions. LW put up with the concept, which uses hydrogen bonds number to represent basepairs. KX put forward the information coefficient formula, which was used in this paper. CK, YZ and JH improved the SPCR program. GW and YW carried out experimental verifications against the result of SPCR program. LZ is responsible for guiding the whole project. All authors have read and approved this final manuscript.

## Supplementary Material

Additional File 1 Zip compressed file, included 4 files in it: SPCR.exe, Help.chm, SimGel.exe, and Cases used to test the SPCR program.mht. SPCR.exe is the main program of SPCR; Help.chm is the help information; SimGel.exe is the assistant program for simulating Gel images; Cases used to test the SPCR program.mht is an html format file, which includes most of detailed data of all cases carried out with SPCR. Click here for file
